# MiR-204 down-regulation elicited perturbation of a gene target signature common to human cholangiocarcinoma and gastric cancer

**DOI:** 10.18632/oncotarget.15290

**Published:** 2017-02-11

**Authors:** Valeria Canu, Andrea Sacconi, Laura Lorenzon, Francesca Biagioni, Federica Lo Sardo, Maria Grazia Diodoro, Paola Muti, Alfredo Garofalo, Sabrina Strano, Antonietta D'Errico, Gian Luca Grazi, Mario Cioce, Giovanni Blandino

**Affiliations:** ^1^ Oncogenomic and Epigenetic Unit, Italian National Cancer Institute ‘Regina Elena', Rome, Italy; ^2^ Faculty of Medicine and Psychology, Surgical and Medical Department of Clinical Sciences, Biomedical Technologies and Translational Medicine, University of Rome ‘La Sapienza', Sant'Andrea Hospital, Rome, Italy; ^3^ HepatoBiliary Pancreatic Surgery, ‘Regina Elena' National Cancer Institute, Rome, Italy; ^4^ Department of Research, Advanced Diagnostic, and Technological Innovation, Regina Elena National Cancer Institute, Rome, Italy; ^5^ Department of Oncology, Juravinski Cancer Center, McMaster University Hamilton, Hamilton, Ontario, Canada; ^6^ Molecular Chemoprevention Group, Italian National Cancer Institute ‘Regina Elena', Rome, Italy; ^7^ Department of Medical and Surgical Sciences, Pathology Unit, S. Orsola-Malpighi Hospital, Alma Mater Studiorum, University of Bologna, Bologna, Italy

**Keywords:** gastric cancer, cholangiocarcinoma, micro-RNA, miR-204, prognostic

## Abstract

**Background & Aims:**

There is high need of novel diagnostic and prognostic tools for tumors of the digestive system, such as gastric cancer and cholangiocarcinoma. We recently found that miR-204 was deeply downregulated in gastric cancer tissues. Here we investigated whether this was common to other tumors of the digestive system and whether this elicited a miR-204-dependent gene target signature, diagnostically and therapeutically relevant. Finally, we assessed the contribution of the identified target genes to the cell cycle progression and clonogenicity of gastric cancer and cholangiocarcinoma cell lines.

**Methods:**

We employed quantitative PCR and Affymetrix profiling for gene expression studies. In silico analysis aided us to identifying a miR-204 target signature in publicly available databases (TGCA). We employed transient transfection experiments, clonogenic assays and cell cycle profiling to evaluate the biological consequences of miR-204 perturbation.

**Results:**

We identified a novel miR-204 gene target signature perturbed in gastric cancer and in cholangiocarcinoma specimens. We validated its prognostic relevance and mechanistically addressed its biological relevance in GC and CC cell lines.

**Conclusions:**

We suggest that restoring the physiological levels of miR-204 in some gastrointestinal cancers might be exploited therapeutically.

## INTRODUCTION

MicroRNAs (miRNAs) are small non-coding RNAs that control gene expression through base pairing with specific sequences in the 3′-UTR of target mRNAs. For their ability to modulate simultaneously the levels of multiple gene products, microRNAs are invaluable tools at integrating intracellular signaling with changes in the extracellular and intracellular milieu, thus conferring spatial and temporal modulation to cell signaling [1]. MicroRNAs may play a role in cancer progression functioning as oncogenes or tumor suppressors [2–5]. In line with this, it is not surprising that specific miRNA signatures have been associated with specific stages of tumor progression, including response to chemotherapy. Consequently, microRNA signatures may be endowed with prognostic and diagnostic potential.

Gastric cancer (GC) is the fifth most frequently diagnosed cancer and the third leading cause of death related to cancer worldwide [6]. The two main histo-types of gastric cancer are the intestinal and the diffuse one [7, 8].

The hepato-biliary tumors are a heterogeneous group of poor prognosis cancers localized to the liver, gall bladder and biliary tree [9]. Establishing the true incidence of bile duct cancer is very difficult because, to date, there are no specific and sensitive diagnostic tools. Based on the location of the tumor cholangocarcinoma can be classified in intrahepatic or extrahepatic cholangiocarcinoma [9, 10].

MicroRNAs, as being exquisite transducers of micro-environmental signaling and sensitive sensors of the tissue homeostasis, can be ideal tools to finely pinpoint the molecular status of a tissue. In facts microRNAs are emerging as effective biomarkers for the differential diagnosis of the tumors, as being proven to discriminate tumoral and normal tissues of various cancer type such as, breast cancers, mesothelioma, head and neck squamous carcinoma and osteosarcomas [11–14].

In a previous study we profiled microRNA differentially expressed in gastric cancer *vs* matched normal tissue specimens and miR-204 was among those differentially modulated in a cohort of 123 matched tissues [14]. MiR-204 was also reported as down regulated in several cancers, including biliary-tree tumors [14–17]. However, little is known about the miR-204 gene targets in various tumors and, more, whether perturbation of miR-204-dependent gene signatures may contribute to tumor progression.

Taking the start from our previous observations, we here evaluated the expression of the miR-204 in a series of tumors of the digestive system and we mechanistically dissected its contribution to tumor progression. Our study hypothesis was that the deep downregulation of the miR-204 in tumor tissues may underlie a very relevant event in the progression of the disease, possibly by triggering gene expression changes, impinging on key protumorigenic properties of the transformed cells. As such, miR-204 levels may be diagnostically and prognostically relevant.

We first aimed at deeply characterize the expression of miR-204 in a sizable cohort of gastric cancer specimens (as compared to normal tissues) to identify, by combining transcription profiling and extensive in silico analysis, the potential targets of this microRNA. This allowed us to identify 37 gene targets with prognostic potential for gastric cancer in Kaplan Meier analysis. Next, we observed that miR-204 down regulation was a rather general feature of other tumors of the digestive system, also including colon, esophageal cancer and hepatocellular carcinoma, which all exhibited a similar degree of miR-204 perturbation. We focused on a rare type of digestive system cancer, the cholangiocarcinoma, because of the availability of a unique large repertoire of specimens in our network. We found that the miR-204 levels, deeply downregulated in cholangiocarcinomas as opposed to normal tissues, correlated with a gene signature almost identical to that observed in gastric cancer specimens. Among the identified gene targets (common to both gastric cancer and cholangiocarcinomas) we identified a 7-gene signature, which exhibited, collectively, prognostic value in Kaplan Meier analysis. We showed that manipulating the levels of miR-204 (by means of agonist molecules) in representative GC and CC cell lines, led to a reversal of the identified gene target signature. This affected the clonogenicity and cell cycle progression of the transfected cell lines, thus establishing a mechanistic link between the modulation of miR-204 targets and the protumorigenic properties of the tumor cells. Finally, we evaluated by siRNA-mediated silencing, the biological activity of each one of the targets toward cell cycle progression and clonogenicity of gastric tumor and cholangiocarcinoma cells.

## RESULTS

**Figure 1:**
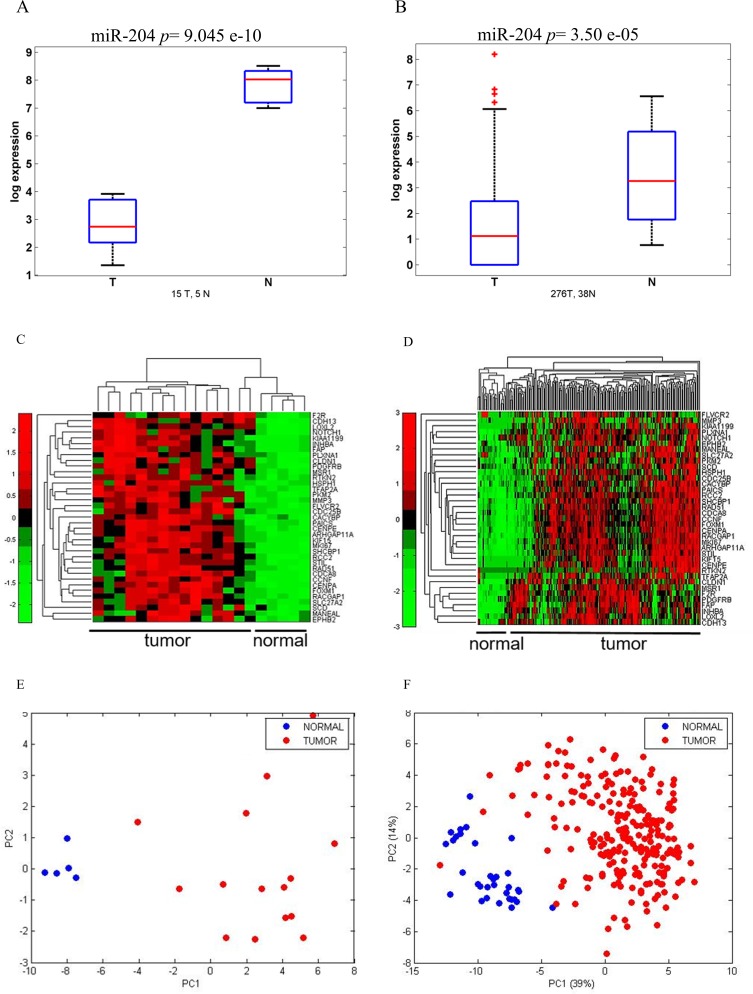
miR 204 and its target gene signature can discriminate between normal and tumoral gastric tissues **A**.-**B**. Boxplots. Expression level of miR-204 in a discovery set of tumor (*n* = 15) and normal (*n* = 5) gastric tissues. Normalized expression levels as assessed by Quantitative PCR. B. Expression levels of miR204 in a validation set of 264 tumoral and 38 normal gastric sample tissues from the TGCA database. **C**.-**D**. Heatmap. Unsupervised hierarchical clustering of the levels of 37 genes targets anti-correlated with miR-204 expression and which contained a miR-204 seed sequence in 3′UTR region (see Table 1) in both the discovery set **C**. and in the TGCA dataset. Normalized intensity values reported. **D**. E-F. Principal Component Analysis (PCA) of the distribution of the 37 genes (as from 1C-1D) in the discovery set **E**. and in the TGCA dataset **F**. The first component (PC1) represents inter-group variability (x-axis) while the second component (PC2) represents the intra-group variability (y-axis).

We recently identified microRNAs differentially modulated in a limited set of matched samples of tumor and normal tissues from gastric carcinoma patients [14]. The microRNA-204 (miR-204) was among the differentially modulated microRNAs and a component of a prognostically relevant signature [14]. To deepen those previous findings, we specifically evaluated the expression of microRNA-204 in a novel discovery set of 20 frozen gastric specimens (15 tumor and 5 normal gastric mucosa) (Table 1), by quantitative PCR (Figure 1A). This showed that the miR-204 levels were much lower in tumor tissues as compared to normal tissues, in line with our previous observations (Figure 1A) [14]. To statistically strengthen this finding, we employed a larger cohort of 276 Tumor and 38 Normal samples from publicly available gene expression databases (TCGA), as a validation set (Table 1) (Figure 1B). Again, we observed similar modulation of the miR-204 in the validation set (Figure 1B). Of note, we also found that a deeper downregulation of the miR-204 levels was a feature of the “intestinal” histotype as opposed to the “diffuse” histotype of gastric cancer (Supplementary Figure 1A). The Intestinal-type gastric cancer is by far the most common variant of gastric cancer (accounting for 50-70% of the cases) (WHO classification of tumours of the digestive system. Lyon: International Agency for Research on Cancer; 2010). Next, we set out to better understand the contribution of miR-204 downregulation to the progression of GC. As microRNAs work mainly by targeting the expression of multiple mRNAs through base paired interactions within their 3′-UTR region, we set out to determine the targets of miR-204 in the tumor tissues analyzed. To do this, we aimed at identifying mRNAs whose expression anti-correlated with that of the microRNA-204 and which contained a microRNA-204 seed sequence in their 3′-UTR region by means of an Affymetrix-based profiling in our discovery set as assessed in Figure 1A (Table 1) (Figure 1C). Extensive *in silico* analysis (Supplementary Figure 1B) allowed us to identify 37 gene targets, which obeyed to the mentioned criteria (Table 3). Indeed, such gene targets, belonging to distinct biological processes (Table 4A-4B), exhibited increased expression in tumoral *vs* normal samples in both our discovery set (Figure 1C, heat map) and in the validation set from TGCA database examined (Figure 1D, heat map). Principal component analysis (PCA) performed on the gene expression levels of the 37 genes in both our discovery set and in the TGCA samples revealed that the expression levels of the microRNA-204 targets could effectively discriminate between tumor and normal samples (Figure 1E, left panel) and between tumor and normal tissue (Figure 1F, right panel), respectively. This was true for both the “intestinal” and “diffuse” histotype of gastric cancer (data not shown). We further refined our analysis and we focused on seven out of 37 identified targets (NOTCH1, FOXM1, RAD51, CENP-A, CENP-E, KIF15, SHCBP1), which showed a significantly high *Pearson anti-Correlation value* in the cohorts analyzed and belonged to similar biological processes (Table 3 and Table 4).

**Table 1:** List of gastric cancer specimens analyzed and clinical information

	DISCOVERY SET	VALIDATION SET (TGCA)	FROZEN TISSUES	KM PLOTTER SET
	*N*	*N*	*N*	*N*
NUMBER OF PATIENTS	20*	220	40	876
SEX				
M	9	140	20	545
F	7	80	20	236
HISTOLOGICAL TYPE				
Diffuse	8	57	10	241
Intestinal	7	140	24	320
Hepatoid	1		2	
Mixed		16		
Not available		7	4	
TUMOR SIZE (T)				
T0			1	
T1	1	9	2	14
T2	2	33	7	241
T3	8	126	16	204
T4	5	52	10	38
Not available				
NODAL STATUS (N)				
N0	2		18	74
N+	14		18	422
Not available			4	
GRADING (G)				
G1	0	26	0	
G2	2	99	17	
G3	10	84	14	
G4	2	11	1	
Unknown	2		8	

Discovery set used for the Affymetrix profiling is composed of 20 samples of which 15 tumoral and 1 un-matched normal gastric mucosa and 4 normal matched gastric mucosae. The validation set is composed of unmatched samples from the TGCA database. Clinical information were available for 220 out of the 314 analyzed patients. Frozen matched samples were used for qPCR validation. KM plotter dataset used for KM analysis. The clinical information available are reported in the table.

**Table 2:** List of cholangiocarcinoma specimens analyzed and clinical information

	FROZEN TISSUES	FFPE TISSUES	TGCA
	*N*	*N*	*N*
NUMBER OF PATIENTS	26	19	36
SEX			
M	17	10	16
F	9	9	20
HISTOLOGICAL TYPE			
intra-hepatic	13	10	30
extra-hepatic	13	8	6
TUMOR SIZE (T)			
T1	2	1	19
T2	12	15	12
T3	7	1	5
T4	1		0
Unknown	3 (biopsy)	2	
NODAL STATUS (N)			
N0	11	10	
N+	12	6	
Unknown	3 (biopsy)	1	
GRADING (G)			
G1	1		19
G2	12	5	9
G3	10	9	1
G4	0		7
Unknown	3 (biopsy)	2	

Frozen matched tissues were characterized for miR 204 and targets genes expression level. FFPE samples were used for studying the miR 204 expression analysis comparing cholangiocarinoma with matched un-affected biliary tract tissues. TGCA database was used to enforce our results.

**Table 3:** Putative miR-204 gene targets selected according to the criteria described in the text

		GENE/miR204 GC		GENE/miR204 TGCA GC		GENE\miR204 TCGA CCA	
GENE	n° prediction tool	R Pearson	*p* value	R Pearson	*p* value	R Pearson	*p* value
RCC2	4	−0,9259432	4,8531E-09	−0,39189627	2,0096E-11	−0,37019042	0,01231233
STIL	4	−0,90967035	2,72938E-08	−0,44694006	1,14003E-14	−0,38264482	0,00948156
MKI67	4	−0,90660095	3,64488E-08	−0,36256828	6,35967E-10	−0,50656626	0,00038426
ARHGAP11A	5	−0,89475864	1,02086E-07	−0,40569912	3,49858E-12	−0,42570867	0,00355126
RAD51	5	−0,89072633	1,41036E-07	−0,31617519	7,6835E-08	−0,43233349	0,00301837
CDCA8	4	−0,88927317	1,57974E-07	−0,28193562	1,62836E-06	−0,49308009	0,00057882
SHCBP1	5	−0,88424802	2,31111E-07	−0,24936578	2,08907E-05	−0,47218798	0,00105717
KIF15	5	−0,88160634	2,80337E-07	−0,38587499	4,20188E-11	−0,42592368	0,00353275
FOXM1	4	−0,86068505	1,11981E-06	−0,28869853	9,18839E-07	−0,52210414	0,00023458
CENPE	5	−0,86029691	1,1465E-06	−0,36510107	4,78314E-10	−0,45798201	0,00155894
CENPA	5	−0,85914302	1,22917E-06	−0,29483137	5,39816E-07	−0,50666915	0,00038304
NOTCH1	6	−0,84324211	3,02813E-06	−0,28549794	1,20686E-06	−0,21394612	0,15818325
RACGAP1	4	−0,84295458	3,07508E-06	−0,3469437	3,49224E-09	−0,50154541	0,00044846
CCNF	5	−0,83267343	5,22975E-06	−0,30009111	3,38701E-07	−0,4391877	0,00254234
PDGFRB	5	−0,82538503	7,46221E-06	−0,21481712	0,000219939	−0,14263023	0,34996411
CDC25B	6	−0,81115812	1,42879E-05	−0,28939643	8,65415E-07	−0,39632472	0,00703537
SLC27A2	6	−0,80642962	1,75228E-05	−0,28327399	1,45574E-06	0,167963321	0,27007769
EPHB2	6	−0,8036086	1,97406E-05	−0,28920466	8,79792E-07	−0,26614462	0,07720508
FLVCR2	5	−0,80297101	2,02742E-05	−0,0582144	0,17304948	0,135952614	0,37320902
PAICS	4	−0,79451311	2,8629E-05	−0,32532083	3,17611E-08	0,038792834	0,80026628
MSR1	5	−0,78443992	4,23269E-05	−0,28314372	1,47174E-06	−0,02154619	0,88827667
PLXNA1	4	−0,78286517	4,49122E-05	−0,31782911	6,56325E-08	−0,32716505	0,02825738
CDH13	4	−0,78204978	4,63035E-05	−0,28911799	8,86364E-07	−0,26173396	0,08242663
CLDN1	5	−0,77870607	5,24052E-05	−0,34912016	2,76994E-09	−0,23370431	0,12232108
HSPH1	7	−0,770814	6,96208E-05	−0,26576022	6,02835E-06	−0,04712768	0,75852626
SCD	5	−0,76457661	8,64784E-05	−0,15310422	0,006377357	0,194194475	0,20116229
RTKN2	7	−0,76044893	9,94697E-05	−0,20430407	0,000420274	−0,3266414	0,0285259
LOXL2	5	−0,75798142	0,00010801	−0,19661312	0,000661776	−0,38198982	0,00961508
CACYBP	5	−0,75630081	0,000114181	−0,39534122	1,30887E-11	−0,39391464	0,00742171
MMP3	5	−0,75226712	0,000130239	−0,16531766	0,003552365	NA	NA
KIAA1199	4	−0,74112207	0,000185051	−0,17897512	0,001762564	−0,23281225	0,12379495
F2R	5	−0,73972529	0,000193143	−0,19232703	0,000846249	−0,07661501	0,61691822
INHBA	7	−0,73433465	0,000227276	−0,27265961	3,48467E-06	−0,18796101	0,21628935
MANEAL	4	−0,7321102	0,000242795	−0,23681267	5,11871E-05	0,036703909	0,81081924
PKM2	4	−0,72843075	0,000270451	−0,21819256	0,000177462	−0,45360692	0,00175125
TFAP2A	4	−0,71306986	0,000416889	−0,24246373	3,43952E-05	−0,48946511	0,00064417
FAP	7	−0,71170461	0,000432668	−0,20515966	0,000399165	−0,2879022	0,0551385

For each gene are reported the number of the microRNA target prediction tools used for the analysis, and the *Pearson anti-Correlation value and the p-value* calculated for our discovery set, for the gastric TGCA dataset, and the cholangiocarcinoma TGCA dataset, respectively.

**Table 4:** A. Gene Ontology of the 37 miR-204 target genes. B. Gene Ontology of the 7 miR-204 target genes

A		
GO terms	*p*-value	NUMBER OF GENES
positive regulation of cellular process	1.92E-5	19
cellular component organization or biogenesis	8.07E-5	19
positive regulation of biological process	9.61E-5	19
developmental process	7.86E-4	18
single-organism developmental process	7.29E-4	17
organelle organization	8.45E-5	13
positive regulation of macromolecule metabolic process	1.49E-4	13
cell cycle process	2.46E-6	11
regulation of cell proliferation	2.11E-4	10
**B**		
**GO terms**	***p*-value**	**GENES**
nuclear division	8,60E+11	cenpa, kif15, cenpe, rad51
mitotic cell cycle process	2,92E-03	cenpa, foxm1, kif15, cenpe
positive regulation of macromolecule metabolic process	7,35E-02	notch1, foxm1, cenpe, rad51
protein-DNA complex assembly	3,05E-03	cenpa, cenpe, rad51
mitotic nuclear division	9,94E-03	cenpa, kif15, cenpe
cellular macromolecular complex assembly	4,86E-02	cenpa, cenpe, rad51
protein complex assembly	9,51E-02	cenpa, cenpe, rad51

**Figure 2:**
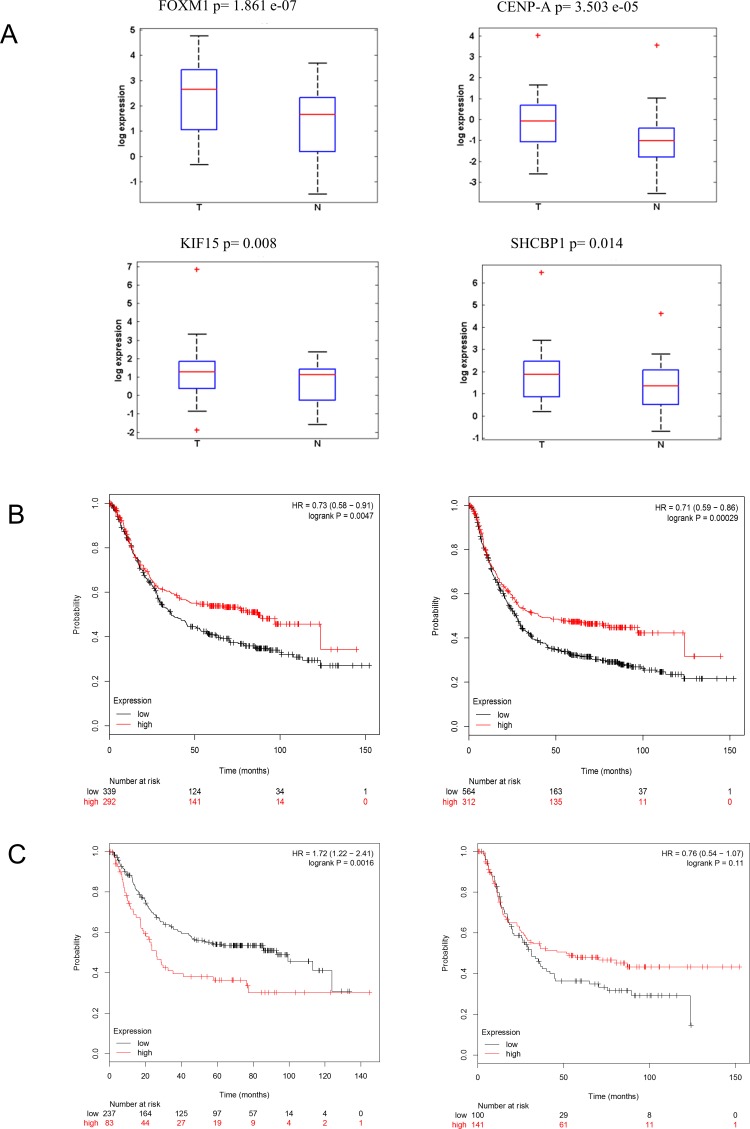
Tumor-expressed miR-204 targets may have prognostic potential **A**. Box plots. Expression levels of CENP-A, SHCBP1, FOXM1, KIF15, in tumor *vs* normal tissue samples as evaluated by quantitative RT-PCR of 41 fresh matched tissues, previously characterized for the miR-204 levels (Supplementary Figure 2A). **B**. Kaplan-Meier analysis. Higher levels of the 37 whole gene signature (left panel) and of the selected 7 gens (FOXM1, RAD51, KIF15, CENP-A, CENP-E, NOTCH1, SHCBP1) (right panel) correlated with decreased patient survival. **C**. Kaplan-Meier analysis. tA subgroup of mir-204 gene targets predicted overall survival of GC patients carrying the intestinal GC histotype. Overall survival calculated on patients bearing intestinal (left panel) or diffuse (right panel) GC histotype, according to the gene set shown in Supplementary Table 1.

We choose to further study the expression of CENP-A, SHCBP-1, FOXM1, KIF15, in a cohort of 40 matched frozen tissue specimens (Table 1) (Figure 2A), previously characterized for the miR-204 levels (Supplementary Figure 2A). We observed that the expression of two out these four genes targets (FOXM1 and KIF15) significantly and positively correlated with the node (N) status of the patient (Supplementary Figure 2B). Next, we evaluated whether the higher levels of the representative genes examined would correlate with patient survival, by Kaplan-Meier analysis (KMplotter.com). This showed that the identified genes contributed prognostic potential, with higher expression of the gene correlating with decreased patient survival. This was true when analyzing both the 37 and the 7 genes signature (Figure 2B left and right panel, respectively).

We also identified a subset of 20 of the 37 genes (including 15 genes common to TGCA and KM plotter datasets) significantly associated to the overall survival of the patients when the latter were stratified according to the tumor histotype, by univariate analysis (Figure 2C left and right panel, Supplementary Table 1). This correlated with a deeper downregulation of miR-204 in the “intestinal” histotype (Supplementary Figure 1A), and with higher levels of its representative candidate targets (Table 5 and Supplementary Figure 5, respectively).

**Table 5:** OZ-ratio and *p*-value of each miR-204 target gene with regard to the tumor histotype (intestinal vs diffuse)

GENE	HISTOTYPE	
	OZ-ratio	p value
**CENPA**	3,524655	8,88E-10
**CDCA8**	3,218823	7,91E-09
**KIF15**	3,872278	9,54E-09
**STIL**	3,194886	1,17E-08
**RACGAP1**	3,569596	2,64E-08
**CENPE**	3,054833	3,76E-08
**SHCBP1**	3,390914	4,79E-08
**FOXM1**	2,801849	4,82E-08
**CCNF**	3,165557	5E-08
**RAD51**	2,871915	1,79E-07
**ARHGAP11A**	2,652069	2,42E-07
**PAICS**	4,033762	4,17E-07
**SCD**	1,910822	9,21E-07
**MKI67**	2,289854	1,04E-06
**PDGFRB**	0,376828	1,32E-06
**CACYBP**	4,86563	1,75E-06
**RCC2**	4,101644	2,01E-06
**CDC25B**	2,151685	3,19E-05
**SLC27A2**	1,636207	6,22E-05
**MANEAL**	1,725689	6,34E-05
**F2R**	0,540483	0,00118
**MMP3**	1,280282	0,002059
**RTKN2**	2,634362	0,002575
**FAP**	0,644902	0,002973
**MSR1**	0,63902	0,004153
**HSPH1**	1,736864	0,004854
**TFAP2A**	1,377954	0,012011
**PKM2**	1,704638	0,015133
**KIAA1199**	1,215815	0,03566
**FLVCR2**	1,526833	0,038384
**EPHB2**	1,212687	0,055602
**CDH13**	0,841145	0,298352
**NOTCH1**	0,835771	0,308333
**INHBA**	0,88429	0,309716
**LOXL2**	1,157181	0,388079
**PLXNA1**	1,174817	0,517301
**CLDN1**	0,947998	0,601849

In bold the significant ones are indicated.

### MiR-204 is downregulated in GI tumors

In order to assess whether the miR-204 downregulation and its consequent alteration of gene transcription would be a gastric cancer specific lesion, we analyzed the levels of expression of miR-204 in matched tissues from other tumors of the digestive system, by employing the TCGA database. This revealed that the miR-204 was downregulated in 103 colon tumor tissues as compared to normal colon mucosa (Supplementary Figure 4A); we also observed that the microRNA-204 was deeply downregulated in 12 esophageal cancer tissues *vs* its normal counterpart (Supplementary Figure 4B) and, finally, albeit with less statistical strength, we found reduced miR-204 expression in 49 hepatic carcinoma tissues (Supplementary Figure 4C).

### Hepatic cholangiocarcinomas exhibited deep miR-204 downregulation and high levels of its target gene signature

**Figure 3:**
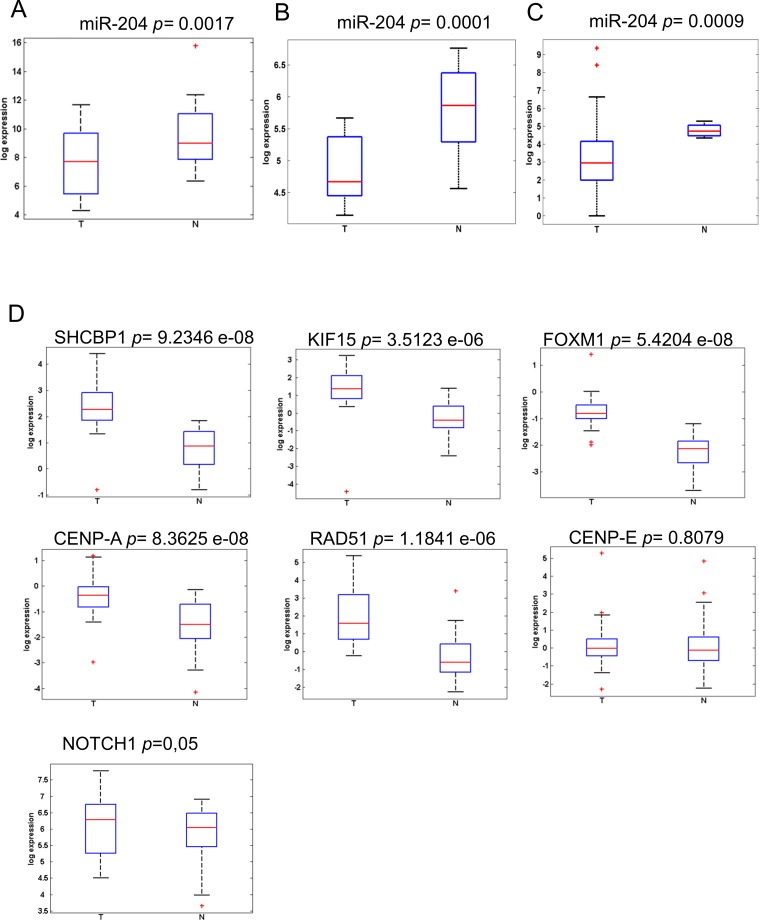
MiR-204 and its target gene signature are differentially expressed in cholangiocarcinoma tissues **A**.-**C**. Expression levels of miR-204 in a discovery set of 26 freshly frozen **A**. and 19 FPPE **B**. cholangiocarcinoma samples as compared to matched normal biliary duct tissues (for the extra-hepatic cholangiocarcinoma) or to hepatic, non tumoral tissues (for the intra-hepatic cholangiocarcinoma), as assessed by quantitative RT-PCR. **C**. Expression levels of miR-204 in a validation set obtained from the TGCA database (*n* = 45). **D**. Expression levels of representative miR-204 target genes as assessed by quantitative PCR from freshly frozen tumor *vs* normal tissues (*n* = 26). CENP-E is not significant upregulated.

**Figure 4:**
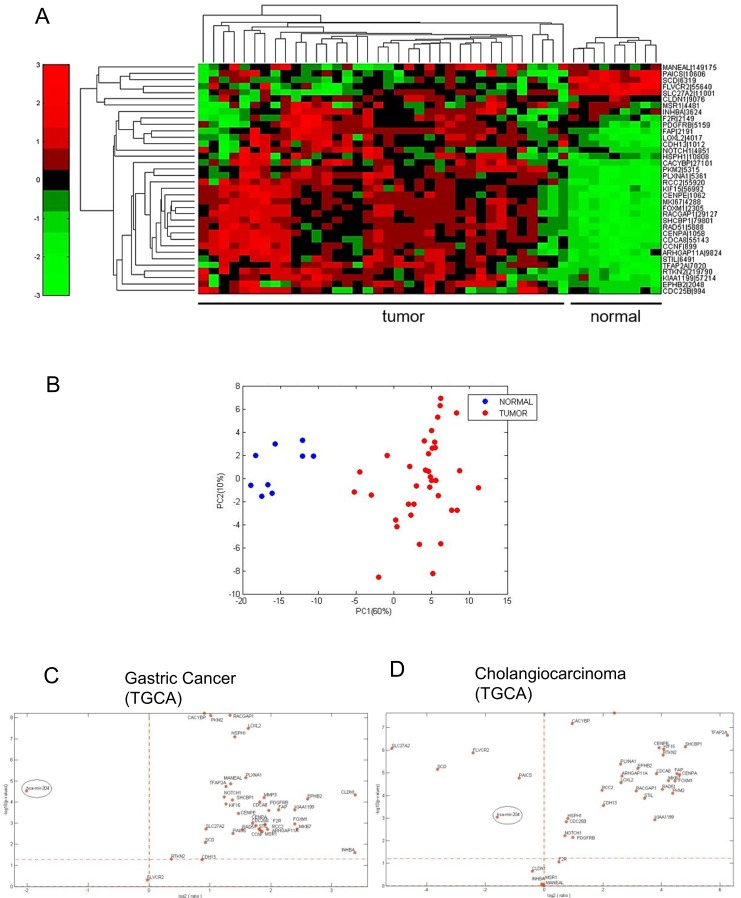
The miR-204 target gene signature is enriched in cholangiocarcinoma tissues **A**. Heatmap. Unsupervised hierarchical clustering of the expression levels of 36 putative miR-204 gene targets (see table 1 please). **B**. Principal Component Analysis (PCA) of the distribution of the 36 genes in the TGCA dataset. **C**. Volcano plots. Analysis of the distribution of the miR-204 levels and those of its putative target genes revealed similar relationship in both gastric cancer and cholangiocarcinoma samples. The log_2_ fold change is plotted on the x-axis and the negative log_10_ of the p-value is plotted on the y-axis.

We evaluated the levels of miR-204 in 26 frozen cholangiocarcinoma tissues and in 19 FFPE samples as opposed to matched normal biliary duct tissues (Table 2). This revealed that the miR-204 was lower in the tumor as opposed to matched normal tissues (Figure 3A). This happened irrespectively whether the RNA was derived from FFPE material or from frozen tissues (Figure 3A-3B). Analysis of the miR-204 expression in 45 cholangiocarcinoma specimens from the TCGA database (Table 2) confirmed our observation (Figure 3C). Next, we assessed the levels of the seven miR-204 gene targets (those already characterized in the gastric cancer specimens). Again, consistently with the prior observation, quantitative PCR of 26 frozen matched cholangiocarcinoma specimens revealed that the 7 genes selected exhibited significantly higher levels in the tumor tissues as opposed to the normal ones (Figure 3D). The analysis of samples in our cohort also showed that the degree of downregulation of the miR-204 and of some of its targets (SHCBP1, CENPA, RAD51) was higher in the intrahepatic cholangiocarcinoma samples as opposed to the extrahepatic ones (Supplementary Figure 5A). We extended the analysis of the levels of the whole set of the previously identified miR-204 targets on the 45 CC samples from TGCA database. This revealed that the selected 36 out 37 targets (expression data for MMP3 was not available) were differentially modulated among tumor and normal tissues derived from the cholangiocarcinoma patients (Figure 4A), again consistently to what observed in the gastric cancer specimens (Table 3). PCA analysis showed that the identified 36-target gene signature could effectively discriminate between tumor and normal tissue (Figure 4B). This was clearly illustrated, for gastric cancer and cholangiocarcinoma, by the Volcano plots in Figure 4C-4D (Figure 4C-4D, respectively) and was true also for esophageal cancer in Supplementary Figure 6 (Supplementary Figure 6). This revealed a high degree of similarity about the modulation of miR-204 and the perturbation of its target genes among three unrelated tumors of the digestive system.

### Restoring miR-204 level in GC and CC cell lines impinged on the expression of common gene targets

**Figure 5:**
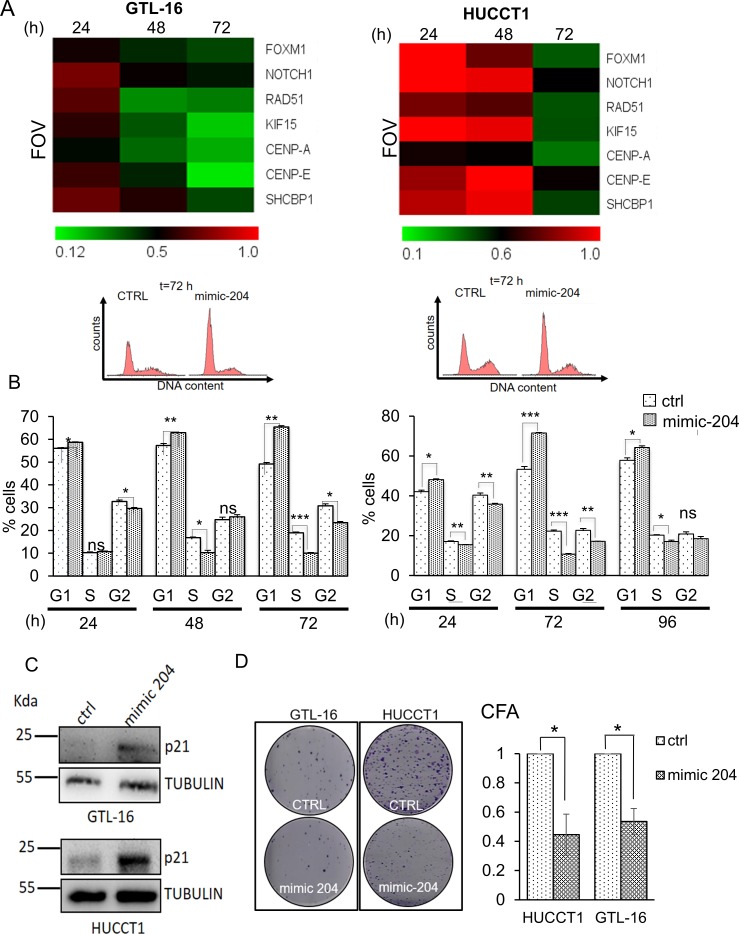
Restoring miR-204 levels in gastric and cholangiocarcinoma cancer cells affects the target signature and some protumorigenic properties of the GTL-16 and HUCCT1 cells **A**. Heat map depicting the expression levels of the indicated miR-204 targets in the GTL-16 and HUCCT1 cell lines transfected with 10 nM of control (ctrl) or mimic-204 and harvested at 24hr, 48hr and 72hr. Normalized expression levels determined by quantitative PCR. The target gene/GAPDH ratio is reported as folds over control. **B**. mimic-204 treatment affects cell cycle. Upper panel. Representative histograms showing the DNA content of GTL-16 (left panel) and HUCCT-1 cells (right panel) stained with propidium iodide at 72 hours after transfection with a control- or the miR-204 agonist. Lower panel. Representative histograms showing the cell cycle distribution of GTL-16 (left panel) and HUCCT-1 cells (right panel) treated as from upper panel. Histogram bars represent the mean ± SE of duplicate experiments. Asterisks: *p < 0,05; **p < 0,01; ***p < 0,001. **C**. Western blotting with p21 antibody of whole cell lysates of GTL-16 (upper panel) and HUCCT1 (lower panel) cells harvested at 72hrs after transfection with a control or a mimic-204. Tubulin staining was used as a loading control. **D**. Clonogenic assays. Left panel. Representative micrographs of colonies formed by the GTL-16 and HUCCT1 cells transfected with a control (ctrl) or a miR-204 agonist molecule (mimic-204) for 96hrs before seeding at clonal density. Histograms bars represent the mean ± SE of triplicate experiments in GTL-16 and HUCCT1 cell lines. Statistics *p* < 0,05 except where indicated.

**Figure 6:**
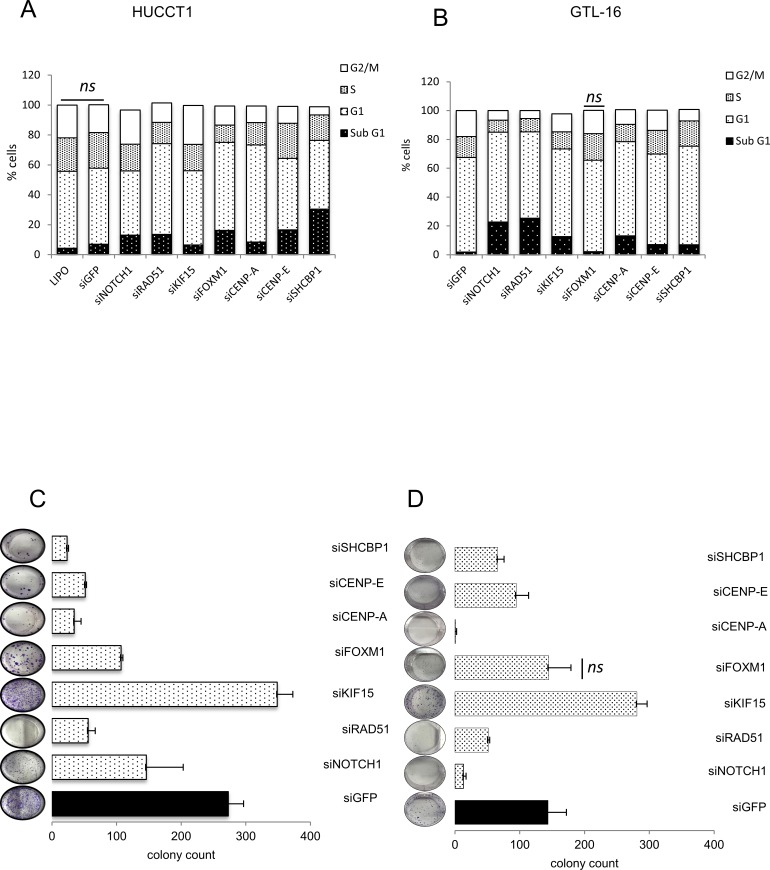
Downregulation of the single miR-204 targets recapitulates the biological effects of miR-204 expression Representative histograms showing the DNA content of HUCCT-1 **A**. and GTL-16 **B**. cells stained with propidium iodide at 72 hrs after transfection with siRNA of the indicated targets. **C**.-**D**. clonogenic assay Representative micrographs of colonies formed by HUCCT1 **C**. and GTL-16 **D**. cells transfected with the indicated siRNA for 72hrs before seeding at clonal density. Histograms bars represent the mean ± SE of triplicate experiments in GTL-16 and HUCCT1 cell lines. Effective silencing was assessed by qPCR (mRNA) data not shown. Statistics *p* < 0,05 except where indicated.

In order to investigate a causal relationship between the lower levels of the miR-204 and the higher levels of its anti-correlated targets, we manipulated the levels of the miR-204 in two representative GC and CC cell lines (GTL-16 and HUCCT1, respectively). We transfected both cell lines with a control (ctrl) or a mimic-204 molecule (mimic-204) and transfection of the latter led to a dose dependent increase of the intracellular levels of the miR-204 (Supplementary Figure 7A). Next, we assessed, by quantitative PCR, the levels of the seven target genes (CENP-A, SHCBP-1, FOXM1, KIF15, CENP-E, RAD51, NOTCH1) at 24hr, 48hr and 72hr from the transfection (10nM). Overexpression of the miR-204 agonist led to time-dependent, decreased expression of the levels of all 7 representative gene targets (Figure 5A, left and right panels). Despite we observed a different kinetics in the GTL-16 as opposed to the HUCCT1 cells transfected with the mimic-204 molecule, in both cases we witnessed effective downregulation of the gene targets, compatible with a reversal of the miR-204 dependent gene expression (Figure 6A, left and right panels, respectively) (*p* < 0.05 at 72hrs). Notably, the effect of transfecting the mimic-204 into both cell lines was conserved even when much lower doses of the agonist were employed, 4nM and 2,5nM respectively (Supplementary Figure 7B-7C) as opposed to10nM-shown in Figure 5A. On the other hand, when we transfected GTL-16 and HUCCT1 cells, stably expressing miR-204 with a miR-204 antagomiR molecule (and a control molecule), we witnessed upregulation of 6 out of the 7 miR-204 targets (Supplementary Figure 8). Next, we evaluated the cell cycle profile of the CTRL- and mimic-204- transfected cells. FACS staining of both GTL-16 and HUCCT1 cells, stained with propidium iodide, revealed a time dependent effect of the agonist treatment on the cell cycle, consisting of an increased G1/S phase, which was maximal at 72hrs (Figure 5B upper and lower panels). We also evaluated, by western blotting of whole cell lysates from GTL-16 and HUCCT1 cells treated with the mimic-204, the expression of the p21 protein, known to increase in cells in G1/S. Compatible with the flow cytometry data, we observed a sharp increase of the p21 protein in the mimic-204 treated cells, as compared to their control counterparts (Figure 5C, left and right panel).

### Altering the miR-204 levels affects clonogenicity

Next, we assessed whether manipulating the levels of the miR-204 gene targets in the GTL-16 and HUCCT1 cells, by means of transfecting mimic-204, could affect some of their protumorigenic properties. To this aim we performed clonogenic assays. This strategy revealed that the clonogenicity of the both cell lines was affected upon overexpression of the miR-204 mimic molecule as compared to the control-transfected cells (Figure 5D). These results are compatible with the previously mentioned involvement of most of the miR-204 candidate targets in the cell cycle control.

### Interference of single miR-204 target genes affects cell cycle and clonogenicity

It is known that the biological effects of the expression of a given microRNA, are mediated by the modulation of clusters of target genes rather than single targets. However, to detail the mechanism of action of the mimic-204, we aimed to investigate which of the miR-204 target gene(s) could mostly influence the miR-204 phenotype. Therefore we transfected the HUCCT1 and the GTL-16 cells with siRNAs targeting each gene of the 7-target signature (Supplementary Figure 9) and we evaluated the effect of the silencing by means of FACS analysis (cell cycle) and clonogenic assays (Figure 6). Staining of the cells with propidium iodide at 72h following siRNA transfection revealed that downregulation of each miR-204 target affected to a varying degree the cell cycle of the transfected cells, compatible with the different biological activities of each of the targets. In detail, silencing of SHCBP1 in HUCCT1 cells strongly increased the Sub-G1 cells (Figure 6A). Similar effects were also consequent to silencing of CENP-E and FOXM1 and for NOTCH1, RAD51 and CENP-A (Figure 6A-6B). Silencing of the mentioned genes affected very similarly both the GTL-16 and HUCCT1 cells, with the exception of FOXM1 in the GTL-16 cells (Figure 6B). Clonogenic assays revealed that silencing of each of the target caused a reduction in colony formation, thus fully matching what observed when ectopically expressing the miR-204 (Figure 6C-6D). Interestingly, silencing of KIF15 did not affect and rather increased the colony forming ability of both the transfected cell lines, despite silencing of the latter elicited increased sub-G1 cells in both cell lines (Figure 6C-6D). Altogether, these observations suggested that increasing the miR-204 intracellular levels triggered effects on the cell cycle and clonogenic ability of the cells which could be partially recapitulated by silencing each of the single miR-204 targets.

## DISCUSSION

In this experimental work we have detailed the modulation of microRNA-204 in both gastric carcinoma and cholangiocarcinoma samples, which exhibited common downregulation of this microRNA in the tumor tissues as opposed to normal tissues. We have identified, in silico, a 37-gene target signature whose levels anti-correlated with those of the miR-204 and shown that 20 out of 37 genes exhibited prognostic potential in GC, with some of the genes of the signature exhibiting independent prognostic potential, in Kaplan-Meier analysis. We have also shown that altering the miR-204 levels in both gastric carcinoma and cholangiocarcinoma cells changed the levels of the miRNA-204 targets genes in a direction compatible with a reversal of the miR-204-dependent effect. Last but not least, exogenously added miR-204 affected the cell cycle progression and inhibited the clonogenicity of the transfected cells. The miR-204 is a well-characterized microRNA, already shown to be downregulated in many solid tumors [15, 18, 19]. Despite the fact that its mode of action and its effector targets may differ according to the tissue of origin and the stage of the tumor, there is indication that it may represent a general tumor suppressor [15, 18–24]. Thus, the data collected here for both GC and CC specimens and the experiments performed in the GTL-16 and HUCCT1 cells are in line with the literature data. This is significant since the collections of specimens analyzed highly differed for their source (FFPE *vs* frozen) and time of collection (TCGA *vs* home-made casuistries) and came from different centers. All these factors could strongly influence the interpatient heterogeneity and contribute significant biases to the analysis.

Notably, 36 out of the identified 37 genes were identically modulated in gastric cancer, cholangiocarcinoma and esophageal cancers tissues as well, thus highlighting the potential relevance of the low levels of the miR-204 observed in GI tumors types. In facts, it is not very common that unrelated tumors exhibit a high degree of similarity in their gene expression profile and this may further suggest a relevant effect of the miR-204 downregulation on the progression of the diseases.

The primary sclerosing cholangitis (PSC) is a chronic liver disease characterized by cholestasis, inflammation and fibrosis and it is known to progress to cholangiocarcinoma (CC) [25, 26]. Recently, a distinct set of microRNAs was identified from sera of patients affected by PSC and CC [27]. Notably, no upregulated miR-204 was observed by Bernuzzi et al. both in the PSC and the CC patient sera. However, it remains to be addressed whether reduced expression of miR-204 would be observed in PSC tissues rather than in patient sera and whether expression of miR-204 may represent a biomarker of progression to CC.

On this line, we found here that the intestinal type of gastric tumors in the TGCA cohorts exhibited a deeper miR-204 downregulation and, consequently, higher levels of its gene target signature. Although the limited data available here do not allow deriving conclusions, it would be tempting in the future to investigate whether our observation may possess patient stratification potential.

Here we partially addressed the specific contribution of each miR-204 gene target to the cell cycle progression and clonogenicity of the GTL-16 and HUCCT1 cells. We found that SHCBP1, RAD51, CENP-A, CENP-E and NOTCH1 were mainly responsible for the “mimic-204 phenotype”. More in detail, while silencing of SHCBP1, RAD51, NOTCH1 and FOXM1 significantly increased the cells in the sub-G1 phase, all of the tested miR-204 targets (except for KIF15) affected the clonogenicity of the transfected cell lines. Downregulation of NOTCH1, among the targets, resulted in effective increase of sub-G1 cells, in line with the NOTCH signaling being a major player in cholangiocarcinoma progression with translational potential for cholangiocarcinoma [28–31].

On the other hand, we did not observe significant induction of sub-G1 cells when transfecting the mimic-204 in both GTL-16 and HUCCT1 cells. Thus, targeting of the single targets did not fully mimic the effect of miR-204 expression. This apparent discrepancy may be explained by the observation that, with regard to the action of the microRNA, it is the coordinated increase of the levels of all or some of its targets that impinges significantly on the overall phenotype. Additionally, *in vitro* experiments revealed that the seven representative target genes exhibited different kinetics of modulation in time. This may be accounted by differences in the steady-state levels of the target mRNAs, different accessibility of the 3′-UTR, or cell subpopulation-specific expression of some targets, within the cell lines examined. With regard to this, we also observed different kinetics between the two cell lines analyzed (GTL-16 and HUCCT1, respectively, with the GTL-16 exhibiting a earlier modulation of the targets when transfected with the mimic-204 (as compared with the HUCCT1 cells, transfected in very similar conditions). This would be, possibly, due to different proliferation rates or intracellular processing of the mimic molecules between the GC and the CC cells.

Last but not least, all of the samples included in our casuistries (both gastric cancer and cholangiocarcinoma) derived from patients not previously treated with chemotherapy. This is an important point. In fact, our and others published work indicate that miR-204 may play a role in determining chemoresistance, at least partially through the modulation of the anti-apoptotic BCL2 protein, in gastric [14] and breast cancer cells [32]. We aim to perform more studies to investigate how the levels of miR-204 are modulated in chemotherapy-treated gastric cancer and cholangiocarcinoma cell lines and, importantly, whether the chemotherapy-induced stress may change the spatial and/or temporal modulation of its targets.

## MATERIALS AND METHODS

### Study population

In the present study 40 gastric surgical specimens from patients who underwent resection for cancer at the Regina Elena Cancer Institute and 11 specimens from the Sant' Andrea Hospital, Rome, were included MiR-204 levels were also analyzed from 26 fresh cholangiocarcinoma tissues of patients who underwent resection for tumor at Regina Elena Cancer Institute and from 19 cholangiocarcinoma formalin embedded tissues (FFPE) from the Sant' Orsola Hospital, Bologna. Case series included also additional hepatic fresh frozen tissues samples, corresponding to 23 HCC and to 11 liver metastases, from Regina Elena Cancer Institute. For each patient, we collected tissue samples from the tumor lesion and from the uninvolved mucosa, defined as the portion of tissue located at 2 cm from the safe resection margin of the tumor lesion. Data regarding patients' demographics, clinical and pathological features as well as the clinical outcomes were obtained from prospectively collected databases. The ethical committees of each of the mentioned institutes have approved the study.

### RNA extraction

Fresh frozen tissues samples were obtained immediately after surgical resection and stored in RNA later (THERMOFISHER, MA USA) to stabilize and protect RNA in fresh specimens. After disruption and homogenization in QIAzol Lysis Reagent (QIAGEN, Chatsworth, CA) RNA was extracted using miRNeasy Kit (QIAGEN Chatsworth, CA) following the manufacturer's instructions. The concentration, purity and quality control of RNA extracted were assessed using Agilent 2100 Bioanalyzer. RNA from FFPE samples was extracted using the miRneasy^®^ FFPE kit (QIAGEN Chatsworth, CA) following the manufacturer's instructions. Total RNA from cell lines was extracted using the TRIZOL Reagents (GIBCO).

### Gene expression profiling

15 tumoral and 5 normal gastric tissues from Regina Elena Cancer Institute and Sant' Andrea Hospital were analyzed for mRNA expression using GeneChip^®^ Human Gene 1.0 ST Array and processed by the Affymetrix Expression Console and normalization.

### cDNA synthesis and qRT-PCR

One microgram of total RNA was reverse-transcribed at 37°C for 60 minute in the presence of random hexamers and Moloney murine leukemia virus reverse transcriptase (Invitrogen). Specific oligonucleotide primers for GAPDH, CENP-A, CENP-E, RAD 51, FOXM1, SHCBP1, KIF15, and NOTCH 1 were used for PCR analyses. GAPDH: FW GAGTCAACGGATTTGGTCGT_RV GACAAGCTTCCCGTTCTCAG; CENP-A: FW GGCCCTATTGGCCCTACAAG_RV GAGTAACTCGGCCTGCATGT; CENP-E: FW GAGGTGCAAAATGCAGGAGC_RV CACATCCTTGCCTGAGGAGG; KIF15: FW AAGCTCCACAAGGAATCCCG_RV ACTCTGGGGTGGTGCTCTAT; SHCBP1: FW CCTGCTTCGAAGGTGACACT_RV TATCAGCACCAGTGCAGTCC; RAD51: FW TGCGACTCGCTGATGAGAGTTT_RV AAACATCGCTGCTCCATCCA; FOXM1: FW TGCAGCTAGGGATGTGAATCTTC_RV GGAGCCCAGTCCATCAGAACT; NOTCH1: FW AGCATCACCTGCCTGTTAGG_ RV TGGCATACACACTCCGAGAA. Gene expression levels were measured by quantitative real-time PCR using the SYBR Green assay (Applied Biosystems, Carlsbad, CA, USA) on a StepOne instrument (Applied Biosystems).

Small amounts of RNA (10 ng) were reverse transcribed using the TaqMan microRNA Reverse Transcription Kit (Applied Biosystems) in a final volume of 10ul using a ABI Prism 7000 Sequence Detection System (Applied Biosystems). The PCR Reactions were initiated with a 10 minute incubation at 95°C followed by 40 cycles of 95°C for 15 seconds and 60°C for 60 seconds. Q-PCR quantification of miRNA expression was performed using TaqMan MicroRNA^®^ Assays (Applied Biosystems) according to the manufacturer's protocol. RNU6B was used as endogenous control to normalize miRNA expression. All reactions were performed in duplicate.

### Cell cultures and treatments

Human gastric cancer cell lineGTL-16 was grown in DMEM medium (Invitrogen) supplemented with 10% fetal bovine serum (FBS), penicillin (100 U/ml) and streptomycin (100ug/ml) at 37°C in a balanced air humidified incubator with 5% CO2. The cholangiocarcinoma cell line HUCCT1 was grown in RPMI medium (Invitrogen) supplemented with 10% fetal bovine serum (FBS), penicillin (100 U/ml) and streptomycin (100ug/ml) at 37°C in a balanced air humidified incubator with 5% CO2.

### Plasmids and transfections

All miRNA mimics, inhibitors, and non-targeting negative controls were purchased from THERMO FISHER (THERMO FISHER MA, USA) and were transfected using Lipofectamine RNAi MAX (Invitrogen) according to the manufacturer's instructions. For mature miR-204-5p expression, we used Pre-miRNA Precursor-Negative Control (THERMO FISHER, MA, USA) and Pre-miRNA204-5p (THERMO FISHER, MA, USA) at final concentration of 10nM, 4nM 2,5nM. For the Inhibition experiments GTL-16 and HUCCT1 stable expressing miR 204 were transfected with antagomiR of miR-204-5p and antagomiR-NC (THERMO FISHER, MA, USA) according to the manufacturer's instructions.

GTL-16 and HUCCT1 cells were transfected with siRNA of NOTCH1, RAD51, CENP-A, CENP-E, FOXM1, KIF15, SHCBP1 for 72 hrs using Lipofectamine RNAi MAX (Invitrogen) according to the manufacture's instructions. siGFP was used as control. Effective silencing was assessed by qPCR (mRNA).

Sequences of siRNA used: RAD51: UGUAGCAUAUGCUCGAGCG; CENPA:CACAGUCGGCGGAGACAAG ; CENP-E: AAGCAGAGAGAAGGGUGAACC; SHCBP1: GCUGAAACUCAUUGAGAAU; KIF15: GCAUGUACAGCUUCAAUUAUU; NOTCH1: AAGUGUCUGAGGCCAGCAAGA; FOXM1: AUAAUUAGAGGAUAAUUUG

### Flow cytometry

Exponentially growing cells were collected at different time points after transfection. Cells were harvested, washed with PBS1x, resuspended in PBS1x/EDTA 5mM, fixed in 70% ETOH/PBS1x and stored at - 20°C. Fixed cells were treated with RNase at final concentration of 1 mg/ml for 30 min at 37°C before addition of 5 mg/ml propidium iodide (PI) (Sigma) and analyzed with Guava Easy-cyte 8HT flow cytometer equipped with Guava Soft 2.1 (Millipore). For each measurement, at least 10,000 cells were acquired.

### Lysate preparation and immunoblotting analyses

Cells were lysed in buffer with 50 mM Tris-HCl pH.8, 150 mM NaCl, 5 mM EDTA, 1% NP-40 (Igepal AC-630). Extracts were centrifuged at 14000 rpm for 10 min to remove cell debris. Protein concentrations were determined by colorimetric assay (Bio-Rad). Western blotting was performed using the following primary antibodies: mouse monoclonal anti-Tubulin (Abcam), rabbit monoclonal anti-p21 (cell signalling). Secondary antibodies used were goat anti-mouse, goat anti-rabbit conjugated to horseradish peroxidase (Amersham Biosciences, Piscataway, NJ, USA). Immunostained bands were detected by chemiluminescent method (Uvitec Alliance, Cambridge).

### Colony-formation assay

1000 live GTL-16 or 500 HUCCT1 cells were transfected with Pre-miRNA Precursor-Negative Control (Ambion) and Pre-miRNA204 (Ambion) before seeding into 6-well dishes (COSTAR). Cells were stained with crystal violet and colonies counted after 7-10 days later.

### Bionformatics and statistical analysis

Signals from gene expression profiling were background adjusted and quantile normalized. A permutation test and a false discovery procedure were used to identify most deregulated genes between tumoral and normal samples, setting to 0.05 the level of significance. Principal component analysis and unsupervised hierarchical clustering were performed on deregulated features. Several prediction target tools were interrogated by using the web server tool MirWalk2 (http://zmf.umm.uni-heidelberg.de/apps/zmf/mirwalk2/) and the most predicted putative targets (at least four prediction software) of miR-204 selected to evaluate miRNA\mRNA correlation. A Pearson's correlation coefficient and relative p-value were obtained for each miRNA-204\mRNA pairs, and genes with p-values less than 0.05 and absolute value coefficient higher than 0.7 were used for further analyses. The obtained signature was first validated in The Cancer Genome Atlas (TCGA) gastric adenocarcinoma and TCGA cholangiocarcinoma dataset, and only genes anti-correlated to miR-204 were selected. A restricted list of genes were further validated by RT-PCR. All the analyses were performed by Matlab (The MathWorks Inc.). For each box-plot shown outlier samples are indicated with asterisks. The survival analysis was performed using web server tool KM plotter. hght and low groups in KM Kaplan-Meier survival curves, was defined by median values of genes expression. Hazard ratio (HR), 95 % confidence intervals (CI), and log-rank p values are also indicated. Signal expression of the signature . aws defined by the mean intensity value.

## SUPPLEMENTARY MATERIALS FIGURES AND TABLES



## References

[1] Donzelli S, Cioce M, Muti P, Strano S, Yarden Y, Blandino G (2016). MicroRNAs: Non-coding fine tuners of receptor tyrosine kinase signalling in cancer. Seminars in cell & developmental biology.

[2] Li MH, Fu SB, Xiao HS (2015). Genome-wide analysis of microRNA and mRNA expression signatures in cancer. Acta pharmacologica Sinica.

[3] Zoni E, van der Pluijm G, Gray PC, Kruithof-de Julio M (2015). Epithelial Plasticity in Cancer: Unmasking a MicroRNA Network for TGF-beta-, Notch-, and Wnt-Mediated EMT. Journal of oncology.

[4] Acunzo M, Romano G, Wernicke D, Croce CM (2015). MicroRNA and cancer—a brief overview. Advances in biological regulation.

[5] Suzuki HI, Katsura A, Matsuyama H, Miyazono K (2015). MicroRNA regulons in tumor microenvironment. Oncogene.

[6] Colquhoun A, Arnold M, Ferlay J, Goodman KJ, Forman D, Soerjomataram I (2015). Global patterns of cardia and non-cardia gastric cancer incidence in 2012. Gut.

[7] Jass JR, Sobin LH, Watanabe H (1990). The World Health Organization's histologic classification of gastrointestinal tumors. A commentary on the second edition. Cancer.

[8] Lauren PA, Nevalainen TJ (1993). Epidemiology of intestinal and diffuse types of gastric carcinoma. A time-trend study in Finland with comparison between studies from high- and low-risk areas. Cancer.

[9] Patel T (2014). New insights into the molecular pathogenesis of intrahepatic cholangiocarcinoma. Journal of gastroenterology.

[10] Bridgewater JA, Goodman KA, Kalyan A, Mulcahy MF (2016). Biliary Tract Cancer: Epidemiology, Radiotherapy, and Molecular Profiling. American Society of Clinical Oncology educational book / ASCO American Society of Clinical Oncology Meeting.

[11] Biagioni F, Bossel Ben-Moshe N, Fontemaggi G, Canu V, Mori F, Antoniani B, Di Benedetto A, Santoro R, Germoni S, De Angelis F, Cambria A, Avraham R, Grasso G (2012). miR-10b*, a master inhibitor of the cell cycle, is down-regulated in human breast tumours. EMBO molecular medicine.

[12] Ganci F, Sacconi A, Manciocco V, Sperduti I, Battaglia P, Covello R, Muti P, Strano S, Spriano G, Fontemaggi G, Blandino G (2016). MicroRNA expression as predictor of local recurrence risk in oral squamous cell carcinoma. Head & neck.

[13] Mori F, Sacconi A, Canu V, Ganci F, Novello M, Anelli V, Covello R, Ferraresi V, Muti P, Biagini R, Blandino G, Strano S (2015). miR-181c associates with tumor relapse of high grade osteosarcoma. Oncotarget.

[14] Sacconi A, Biagioni F, Canu V, Mori F, Di Benedetto A, Lorenzon L, Ercolani C, Di Agostino S, Cambria AM, Germoni S, Grasso G, Blandino R, Panebianco V (2012). miR-204 targets Bcl-2 expression and enhances responsiveness of gastric cancer. Cell death & disease.

[15] Chen X, Liu XS, Liu HY, Lu YY, Li Y (2016). Reduced expression of serum miR-204 predicts poor prognosis of gastric cancer. Genet Mol Res.

[16] Tong HX, Zhou YH, Hou YY, Zhang Y, Huang Y, Xie B, Wang JY, Jiang Q, He JY, Shao YB, Han WM, Tan RY, Zhu J (2015). Expression profile of microRNAs in gastrointestinal stromal tumors revealed by high throughput quantitative RT-PCR microarray. World journal of gastroenterology.

[17] Zhang B, Yin Y, Hu Y, Zhang J, Bian Z, Song M, Hua D, Huang Z (2015). MicroRNA-204-5p inhibits gastric cancer cell proliferation by downregulating USP47 and RAB22A. Medical oncology.

[18] Wu X, Zeng Y, Wu S, Zhong J, Wang Y, Xu J (2015). MiR-204, down-regulated in retinoblastoma, regulates proliferation and invasion of human retinoblastoma cells by targeting CyclinD2 and MMP-9. FEBS letters.

[19] Ying Z, Li Y, Wu J, Zhu X, Yang Y, Tian H, Li W, Hu B, Cheng SY, Li M (2013). Loss of miR-204 expression enhances glioma migration and stem cell-like phenotype. Cancer research.

[20] Wang L, Tian H, Yuan J, Wu H, Wu J, Zhu X, CONSORT (2015). Sam68 Is Directly Regulated by MiR-204 and Promotes the Self-Renewal Potential of Breast Cancer Cells by Activating the Wnt/Beta-Catenin Signaling Pathway. Medicine.

[21] Wu ZY, Wang SM, Chen ZH, Huv SX, Huang K, Huang BJ, Du JL, Huang CM, Peng L, Jian ZX, Zhao G (2015). MiR-204 regulates HMGA2 expression and inhibits cell proliferation in human thyroid cancer. Cancer biomarkers : section A of Disease markers.

[22] Yin Y, Zhang B, Wang W, Fei B, Quan C, Zhang J, Song M, Bian Z, Wang Q, Ni S, Hu Y, Mao Y, Zhou L (2014). miR-204-5p inhibits proliferation and invasion and enhances chemotherapeutic sensitivity of colorectal cancer cells by downregulating RAB22A. Clin Cancer Res.

[23] Xia Y, Zhu Y, Ma T, Pan C, Wang J, He Z, Li Z, Qi X, Chen Y (2014). miR-204 functions as a tumor suppressor by regulating SIX1 in NSCLC. FEBS letters.

[24] Qiu YH, Wei YP, Shen NJ, Wang ZC, Kan T, Yu WL, Yi B, Zhang YJ (2013). miR-204 inhibits epithelial to mesenchymal transition by targeting slug in intrahepatic cholangiocarcinoma cells. Cellular physiology and biochemistry.

[25] Bergquist A, Ekbom A, Olsson R, Kornfeldt D, Loof L, Danielsson A, Hultcrantz R, Lindgren S, Prytz H, Sandberg-Gertzen H, Almer S, Granath F, Broome U (2002). Hepatic and extrahepatic malignancies in primary sclerosing cholangitis. Journal of hepatology.

[26] Burak K, Angulo P, Pasha TM, Egan K, Petz J, Lindor KD (2004). Incidence and risk factors for cholangiocarcinoma in primary sclerosing cholangitis. The American journal of gastroenterology.

[27] Bernuzzi F, Marabita F, Lleo A, Carbone M, Mirolo M, Marzioni M, Alpini G, Alvaro D, Muri Boberg K, Locati M, Torzilli G, Rimassa L, Piscaglia F (2016). Serum micrornas as novel biomarkers for primary sclerosing cholangitis and cholangiocarcinoma. Clinical and experimental immunology.

[28] El Khatib M, Bozko P, Palagani V, Malek NP, Wilkens L, Plentz RR (2013). Activation of Notch signaling is required for cholangiocarcinoma progression and is enhanced by inactivation of p53 *in vivo*. PloS one.

[29] Gil-Garcia B, Baladron V (2016). The complex role of NOTCH receptors and their ligands in the development of hepatoblastoma, cholangiocarcinoma and hepatocellular carcinoma. Biology of the cell.

[30] Razumilava N, Gores GJ (2013). Notch-driven carcinogenesis: the merging of hepatocellular cancer and cholangiocarcinoma into a common molecular liver cancer subtype. Journal of hepatology.

[31] Zender S, Nickeleit I, Wuestefeld T, Sorensen I, Dauch D, Bozko P, El-Khatib M, Geffers R, Bektas H, Manns MP, Gossler A, Wilkens L, Plentz R (2013). A critical role for notch signaling in the formation of cholangiocellular carcinomas. Cancer cell.

[32] Wang X, Qiu W, Zhang G, Xu S, Gao Q, Yang Z (2015). MicroRNA-204 targets JAK2 in breast cancer and induces cell apoptosis through the STAT3/BCl-2/survivin pathway. International journal of clinical and experimental pathology.

