# Supplementary material for: MiR-204 down-regulation elicited perturbation of a gene target signature common to human cholangiocarcinoma and gastric cancer
Source: Oncotarget. 2017 Feb 11;8(18):29540–57. doi: 10.18632/oncotarget.15290 (PMC5444686; doi:10.18632/oncotarget.15290)
Supplement: Supplementary file 1 [file oncotarget-08-29540-s001.pdf]

MiR-204 down-regulation elicited perturbation of a gene target signature common to human cholangiocarcinoma and gastric cancer

Supplementary Material

A

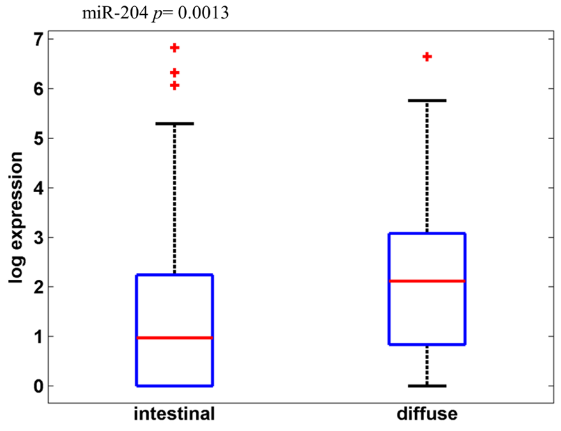

B

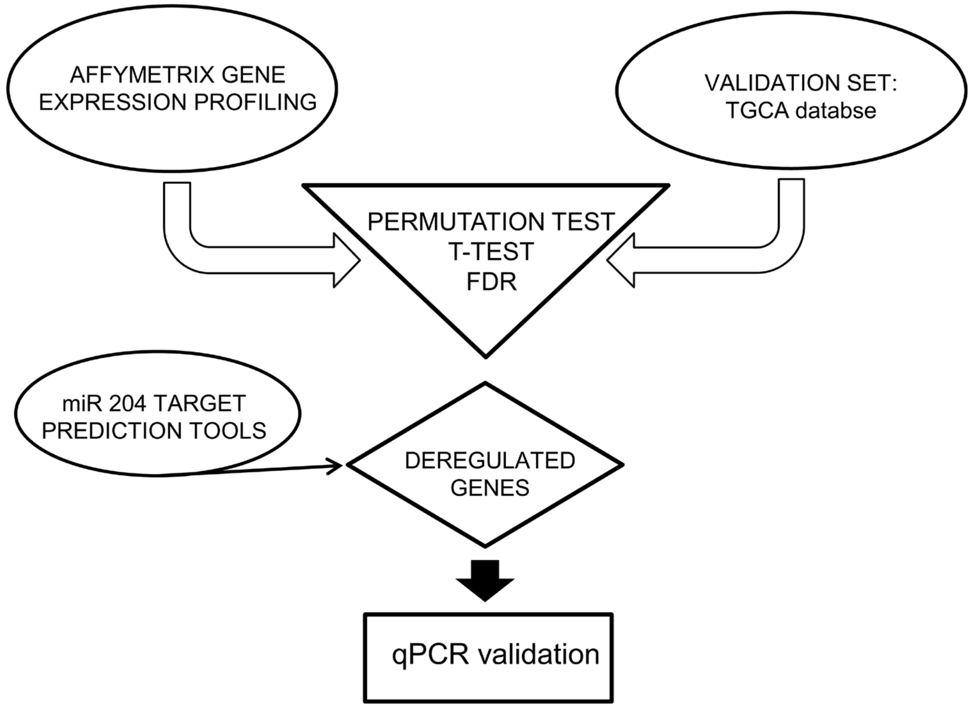

Supplementary Figure 1: miR-204 shows deeper downregulation in the intestinal histotype of gastric cancer. A Box-plot. miR-204 levels in cohorts of 140 intestinal and 57 diffuse gastric cancer. Data obtained from the validation set (TGCA database). B Schematic workflow of *in silico* analysis performed in this study to identify putative miR-204 targets.

A

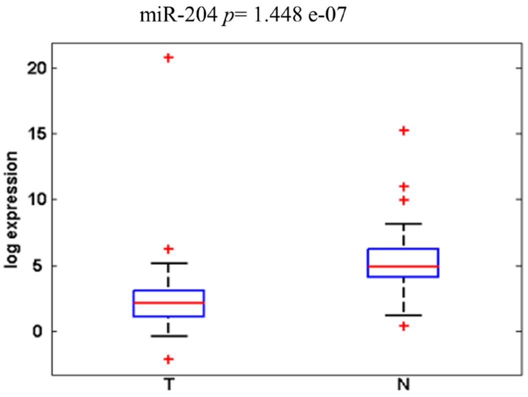

B

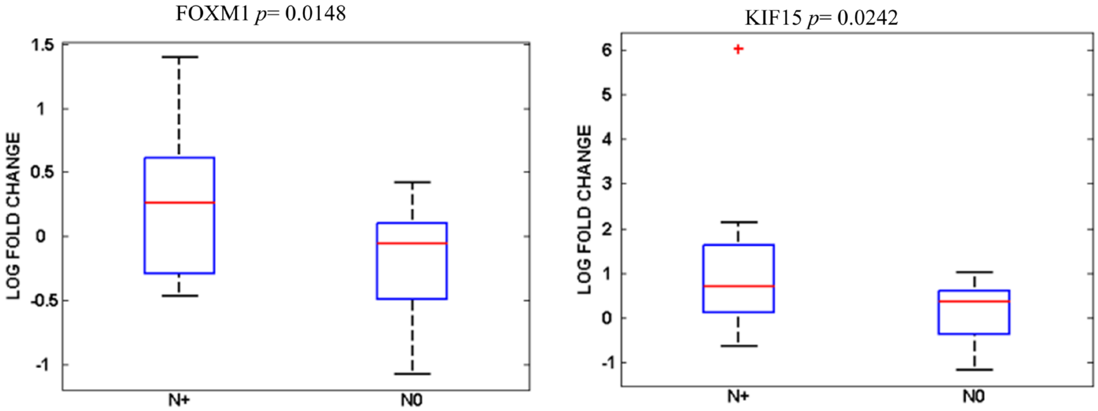

Supplementary Figure 2: miR 204 is strongly downregulated in gastric cancer tissues. A. Box-plots. miR-204 levels in our collection of 41 freshly frozen gastric tumors compared to matched normal tissues as evaluated by quantitative PCR. B. FOXM1 and KIF15 levels correlate with the N status of gastric cancers. Box-plots. FOXM1 and KIF15 expression levels in 41 freshly frozen tumor tissues from node positive (N+) and node negative (N0) gastric cancer patients. Quantitative PCR.

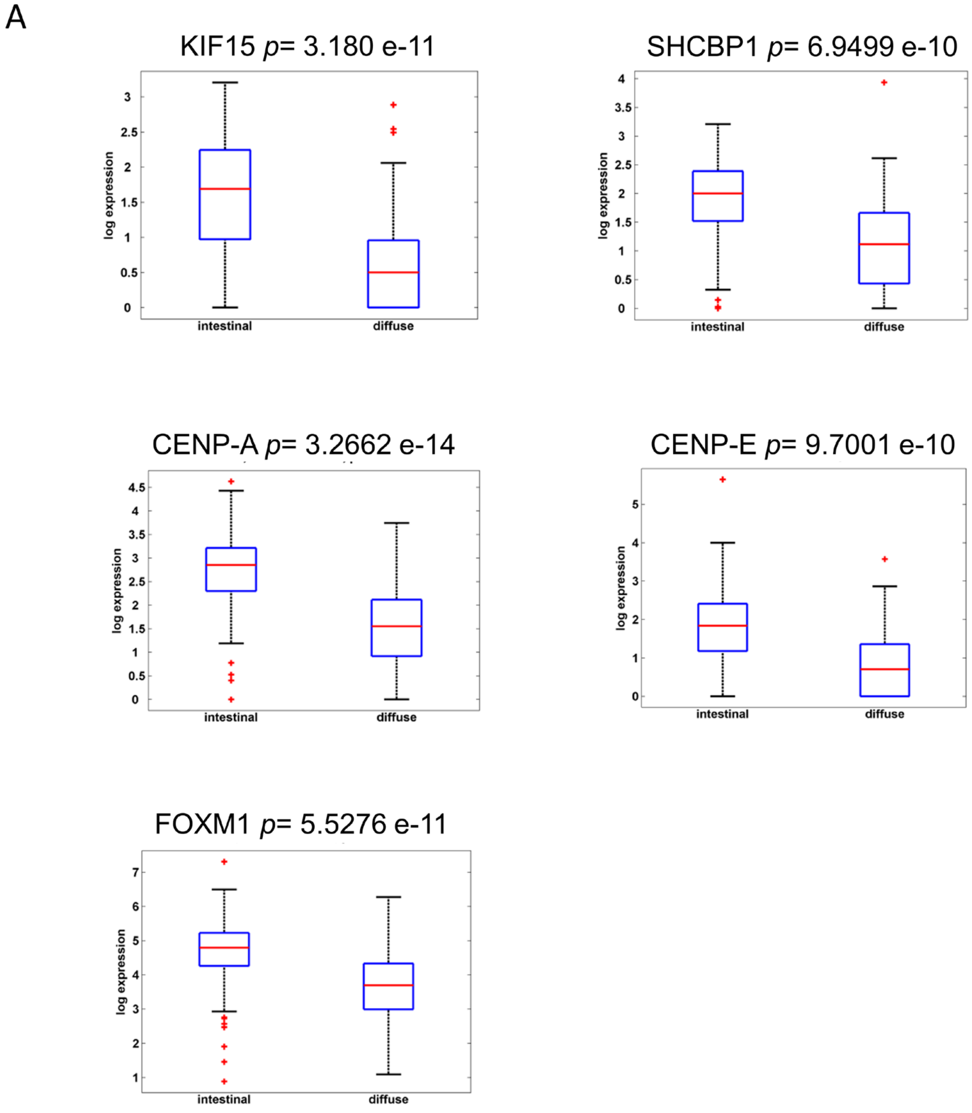

Supplementary Figure 3: The miR-204 target genes are differentially expressed in intestinal vs diffuse GC. Box-plots showing the levels of representative miR-204 target genes in intestinal vs diffuse gastric cancers.

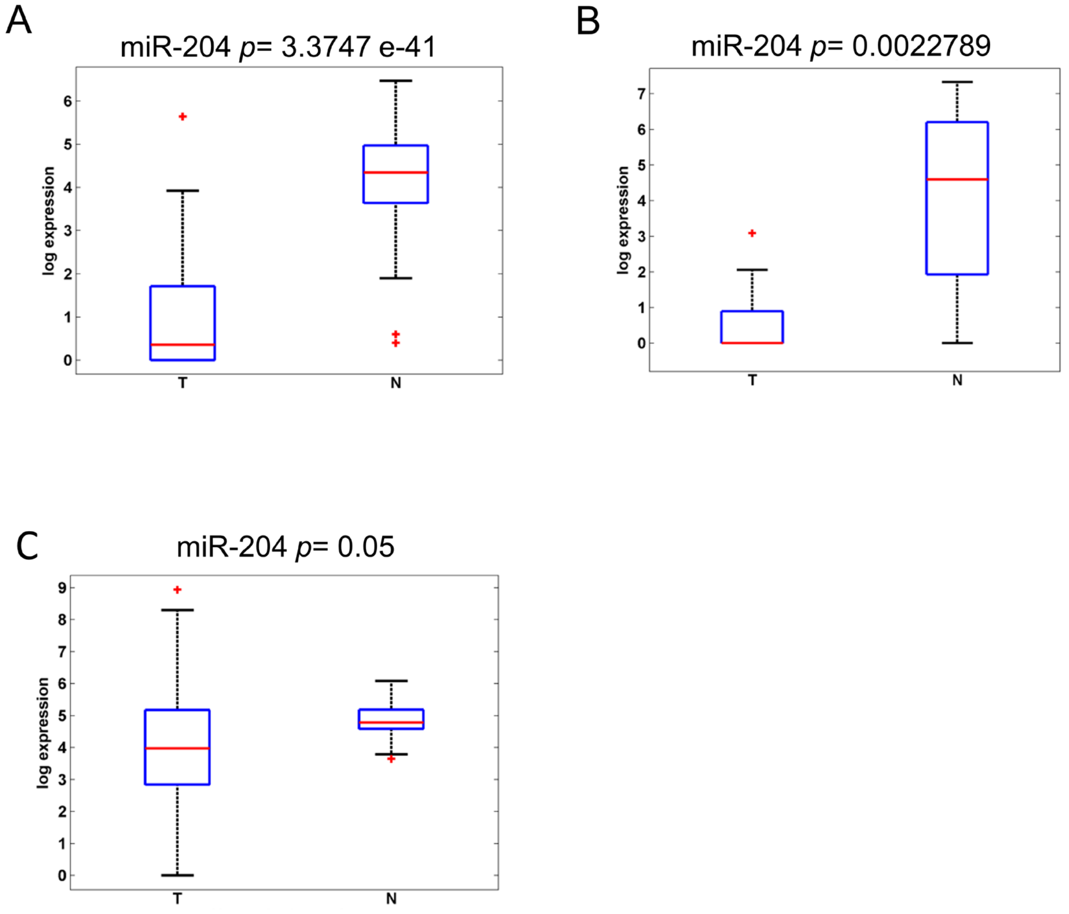

Supplementary Figure 4: miR-204 is significantly downregulated in unrelated tumors of the digestive system. A-C Box-plot miR-204 level in a cohort of matched normal and tumor tissues of colon cancer (n=103) (A), esophageal cancer (n=12) (B), and hepatocellular carcinoma (n=49) (C) from TGCA database.

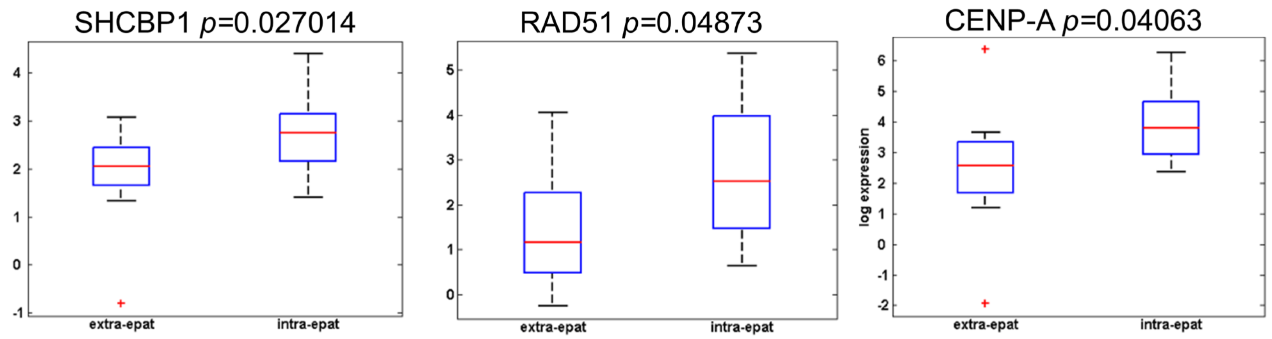

Supplementary Figure 5: miR-204 target genes expression in intrahepatic and extrahepatic cholangiocarcinoma A. Box-plot. Expression levels of the SHCBP1, CENP-A and RAD51 mRNAs in intrahepatic and extrahepatic cholangiocarcinoma tissues.

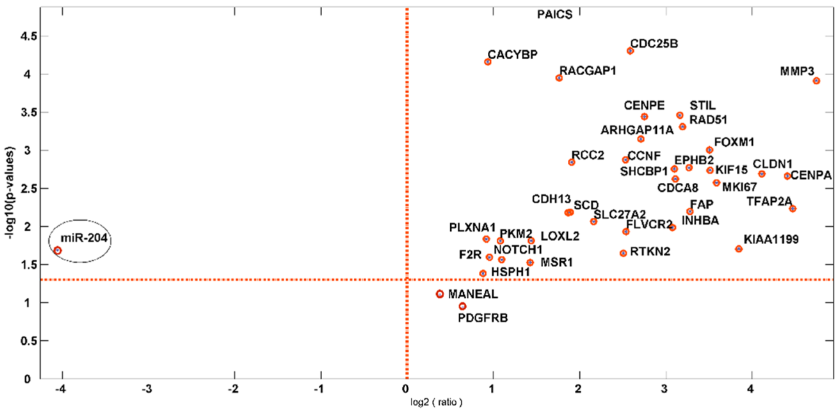

Supplementary Figure 6. Volcano plot analysis of the distribution of miR-204 levels and its putative target genes in matched esophageal cancer vs normal tissues from TGCA database. The $\log_2$ fold change is plotted on the x-axis and the negative $\log_{10}$ of the p-value is plotted on the y-axis.

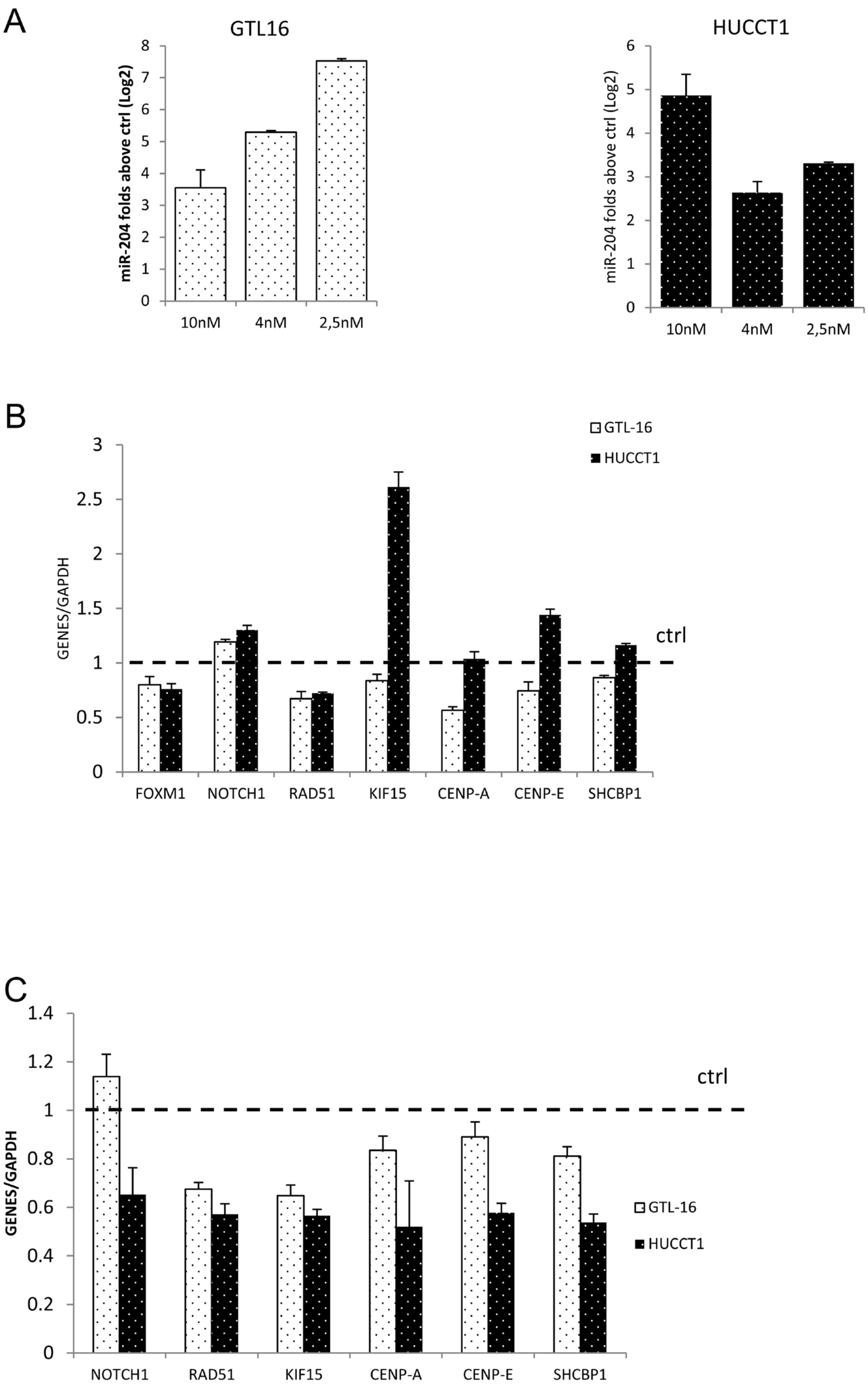

Supplementary Figure 7: Restoring miR-204 levels in gastric cancer and cholangiocarcinoma cell lines affected its target signature and the clonogenicity of the GTL-16 cells. A. Histograms depicting the expression levels of miR-204 as assessed by quantitative PCR from GTL16 cell lines (left panel) and HUCCT1 (right panel) transfected with the indicated doses of control (ctrl) or a miR-204 agonist molecule (mimic-204) and harvested for RNA extraction 48hr later. B. Quantitative PCR histograms depicting the expression levels of selected miR-204 targets in the GTL16 cell lines and HUCCT1 transfected with 4nM (B) and 2,5nM (C) of control (ctrl) or mimic-204. Dashed lines represent the levels of the targets in control-transfected cells. Please note that the expression levels of the cells transfected with 10nM of ctrl or mimic-204 are reported in Fig. 5.

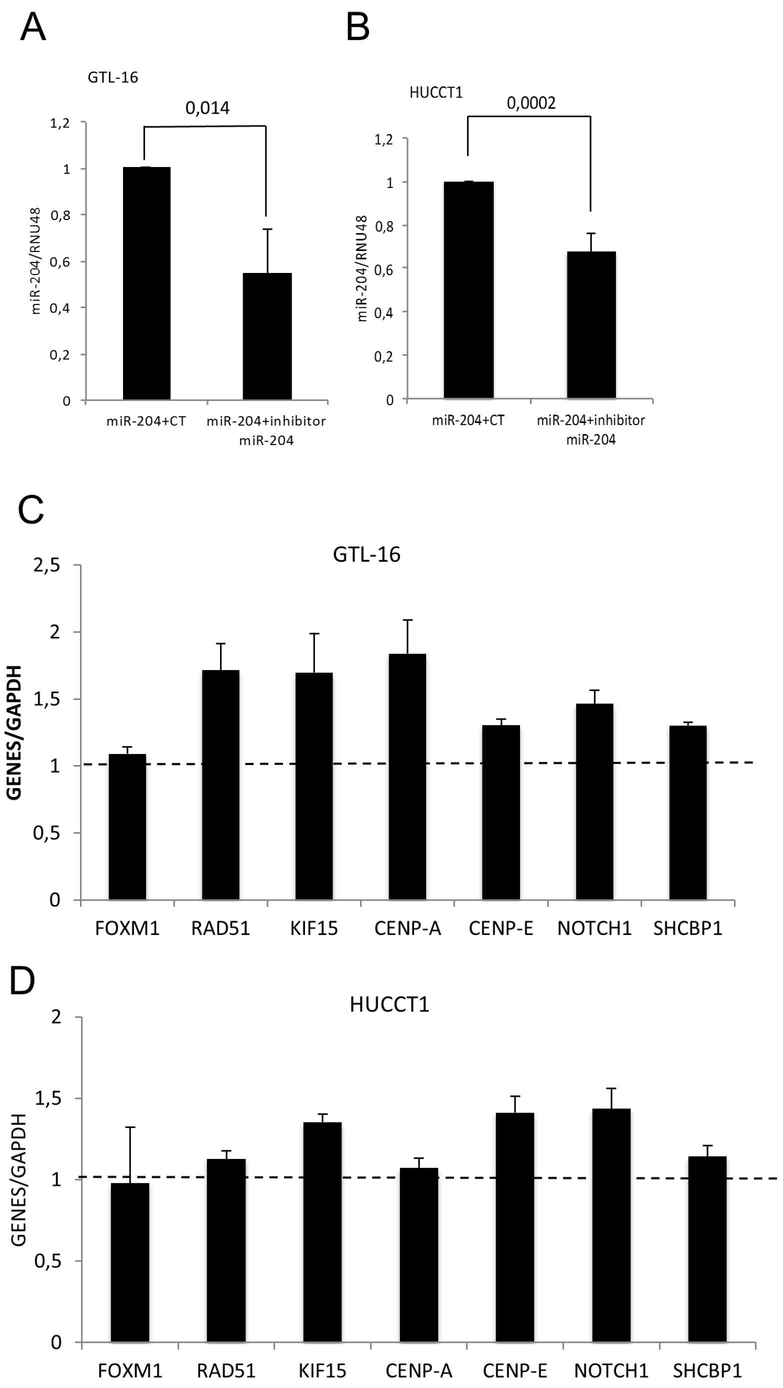

Supplementary Figure 8:Transfection of GTL-16 and HUCCT1 stably overexpressing miR-204 with a synthetic inhibitor reduced miR-204 expressions and relieved repression of its targets. A. Quantitative PCR. Expression level of miR-204 in GTL-16 stably overexpressing miR-204 48h after inhibitor transfection. B Expression level of miR-204 in HUCCT1 stably overexpressing miR-204 48h after inhibitor transfection. C-D. Transfection of GTL-16 stable overexpressing miR-204 with a synthetic inhibitor of miR-204 upregulated its identified targets. Expression levels of the indicated miR-204 gene targets in GTL-16 and HUCCT1 cells (stably expressing the miR-204) transfected with mir-204 antagomiR and a control, respectively. The dashed line indicates folds over control. The bars report the median value of three independent experiments.

A

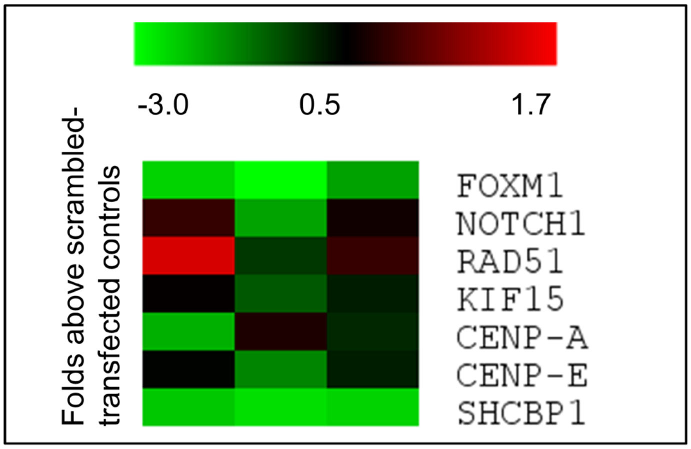

B

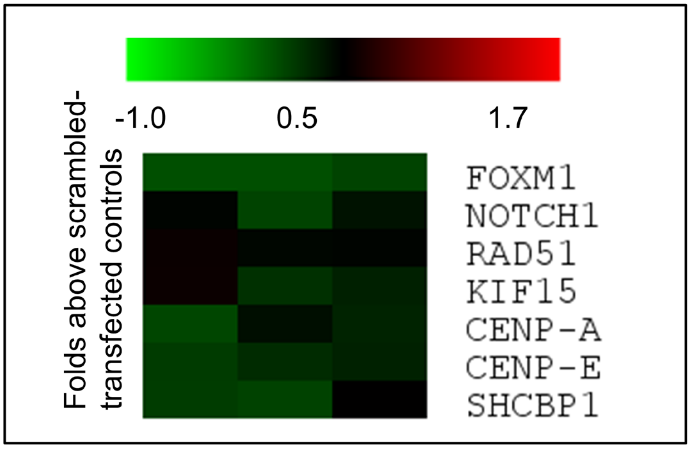

Supplementary Figure 9: Downregulation of the miR-204 target mRNAs in GTL16 and HUCCT1 cells transfected with specific siRNAs. A-B Heat-map Expression level of single miR-204 target genes after 72h with siRNA transfection (as compared to scramble si-RNAs) in GTL-16 (A) and HUCCT1(B), respectively.

Supplementary Table 1: A list of the 20-gene target set used for Overall survival analysis in fig. 2C.

1	STIL
2	MKI67
3	FOXM1
4	CENP-A
5	RACGAP1
6	FBX1
7	PDGFRB
8	CDC25B
9	EPHB2
10	PAICS
11	MSR1
12	PK3
13	TFAP2A
14	NOTCH1
15	RAD51
16	SHCBP1
17	KIF15
18	CENPE
19	PLXNA1
20	CDH13
